# Design of Ready-to-Use “Ball-in-Ball” *Staphylococcus aureus* Microsphere Based on Novel Cryoprotectant and Drop Freeze-Drying Technology: Effective Preservation and Application

**DOI:** 10.3390/foods14122142

**Published:** 2025-06-19

**Authors:** Zile Wang, Dongdong Chen, Xiaomei Zheng, Yuqing Li, Shaoqian Jiang, Yanfei Chen, Jingjian Jia, Libo Yu, Tao Peng

**Affiliations:** 1 Chinese Academy of Quality and Inspection & Testing, Beijing 100176, Chinajiangsq@acas.com.cn (S.J.);; 2 Eberly College of Science, The Pennsylvania State University, University Park, PA 16802, USA; zxt0616@163.com

**Keywords:** foodborne pathogens, response surface design, cryoprotectant, ready to use, freeze-dried survival rate

## Abstract

*Staphylococcus aureus* (*S. aureus*) poses a significant threat to public health and safety, and enhancing the monitoring of *S. aureus* in food is essential to curb and prevent foodborne transmission. In order to obtain strains for more convenient and rapid use in quality control or quantitative analysis, this study designed a ready-to-use “ball-in-ball” microsphere based on a novel cryoprotectant combined with drop freeze-drying technology. When using a cryoprotectant that contains 1.5% bovine serum albumin, 4.5% trehalose, 8.2% polyethylene glycol 8000, and 4.1% D-mannitol, the survival rate of *S. aureus* can reach 98.2 ± 2.6%. This cryoprotectant effectively prevents *S. aureus* from shrinking, deforming, and damaging cell walls. Additionally, it shows desirable protective efficiency for other Gram-positive bacteria. The molding of microspheres is efficient and cost-effective, demonstrating good uniformity and stability without the need for pre-freezing. The moisture content and the count of *S. aureus* showed no significant changes over 90 days at −20 °C. In the simulated contaminated sample, the recovery rate of *S. aureus* in milk and green tea was 83.1–93.7%. This study could provide a practical approach to improve the monitoring efficiency of *S. aureus* and shows potential as a generalized strategy for preparing ready-to-use strains related to food safety.

## 1. Introduction

Foodborne pathogens, particularly harmful bacteria, represent a serious threat to public health and pose a critical global challenge [1]. They not only cause illnesses but also cleverly exploit food as a means of transmission [2]. *Staphylococcus aureus* (*S. aureus*) is a significant foodborne pathogen that can secrete toxins such as enterotoxins and toxic shock syndrome toxin-1, leading to vomiting, gastroenteritis, diarrhea, or toxic shock syndrome [3]. It is also responsible for a significant number of skin infections, pneumonia, nosocomial bacteremia, and cardiovascular infections [4]. *S. aureus* is widely distributed in nature, so enhancing the monitoring of *S. aureus* in food is essential to curb and prevent foodborne transmission [5].

There are various techniques available for detecting *S. aureus*, including traditional separation and cultivation, molecular biology technology, biosensors, immunoassays, etc. Traditional separation and cultivation are regarded as the gold standard [6]; however, the detection processes are often lengthy and cumbersome, making it challenging to achieve rapid on-site results. Rapid detection, including immunoassays and biosensors, significantly enhance detection efficiency in batch sample analysis. For instance, Jiang et al. developed a surface-enhanced Raman scattering (SERS) biosensor for *S. aureus*, *Pseudomonas aeruginosa* and *Escherichia coli* (*E. coli*) O157:H7, allowing the on-site analysis less than 50 min [7]. Yang et al. developed a one-step electrochemical sensor for *S. aureus*, *E. coli*, and *Salmonella typhimurium*, and the fully integrated detection strategy enables a rapid quantification of target pathogens in 20 min [8]. Albashir et al. proposed a novel paper biosensor for on-spot and rapid detection of *S. aureus*, demonstrating remarkable selectivity and high sensitivity, with the entire detection process taking only 60 min [9]. Dai et al. constructed a colorimetric-SERS by using MnO_2_@AuNPs nanozyme-linked aptamer to replace the traditional enzyme-linked immunosorbent assay for the detection of *S. aureus*, achieving results in approximately 60 min [10]. However, although the analysis process has been effectively shortened, it is still unavoidable to culture *S. aureus* overnight (18–24 h) for quality control or quantitative analysis before the analysis process [7,9], which creates a bottleneck that hinders the improvement of detection efficiency. A ready-to-use strain with a known quantity that does not require pre-culturing would be extremely beneficial.

Freeze-drying is one of the most effective methods for preserving bacterial strains. However, during the process, bacteria are susceptible to various stresses such as low temperatures, osmotic pressure, and dehydration [11]. These conditions likely affect cell membrane fluidity, compromise membrane integrity, disrupt pH balance, and decrease intracellular enzyme activity [12], ultimately leading to bacterial death. Previous research has shown that incorporating cryoprotectants during freeze-drying significantly enhances the survival rate [13]. In addition, relying on a single type of protective agent often does not yield optimal protective effects. As a result, researchers typically combine multiple protective agents to achieve better outcomes. Chen et al. developed a petroleum hydrocarbon-degrading bacterial strain powder using a cryoprotectant containing skim milk powder, trehalose, sucrose, and glycerol to enhance the bioremediation of polluted soil, and the bacterial survival rate reached as high as 95% [14]. Li et al. developed an effective strategy to improve the freeze-drying survival rate of *Lactobacillus curvatus*. By using a cryoprotectant that contains trehalose, oleic acid, and skim milk, the survival rate reached 85.38% [11]. Research on freeze-drying strategies primarily focuses on probiotics, with limited studies conducted on foodborne pathogens. The effective freeze-drying strategies for *S. aureus* require further development, and identifying a more convenient and cost-effective form of strain could represent a new breakthrough.

To obtain strains for more convenient and rapid use in quality control or quantitative analysis, based on the similar shapes of *S. aureus* and the microsphere, this study designed a ready-to-use strain named the “ball-in-ball” microsphere. A novel composite cryoprotectant was developed by combining single-factor evaluations and response surface design. The impact of various protective agents on the survival rate of *S. aureus* and physical appearance of the microsphere was discussed in detail. Compared to the commonly used powder preservation technology, drop freeze-drying was employed to shape the liquid bacteria [15], making it convenient and cost-effective. The parameters of the microsphere were optimized, and its moisture content, stability, uniformity, and solubility were assessed. In order to verify the availability of the developed *S. aureus* microspheres in actual food sample detection, the recovery rates in milk and green tea were assessed. This study could provide a theoretical foundation and technical support for the rapid and efficient detection of foodborne pathogens.

## 2. Materials and Methods

### 2.1. Materials

*S. aureus* (ATCC 25923), *E. coli* (ATCC 8739), *Salmonella* (ATCC 13076), *Listeria monocytogenes* (ATCC 19111), *Shigella sonnei* (ATCC 9290), *Bacillus cereus* (ATCC 33019), *Cronobacter muytjensii* (ATCC 51329), *Klebsiella pneumoniae* (ATCC 13883), and methicillin-resistant *S. aureus* (ATCC 333591) were stored in our laboratory. Nutrient agar and brain heart infusion agar were purchased from Beijing Land Bridge Technology Co., Ltd. (Beijing, China). D-Mannitol and gelatin were purchased from Merck (Darmstadt, Germany). α-lactose was purchased from Sinopharm Chemical Reagent Co., Ltd. (Shanghai, China). D(+)-Trehalose dihydrate (TRE) was purchased from Tokyo Chemical Industry (Tokyo, Japan). L-(+)Sodium glutamate (GLU) was purchased from Adamas-beta (Shanghai, China). Skim milk powder (SMP) was purchased from Becton, Dickinson and Company (Franklin Lakes, NJ, USA). Arabic gum (AG) was purchased from Shanghai yuanye Bio-Technology Co., Ltd. (Shanghai, China). Dextran was purchased from Shanghai Macklin Biochemical Technology Co., Ltd. (Shanghai, China). Betaine, polyethylene glycol (PEG) 20000, PEG 8000, tween 40, glycine, bovine serum albumin (BSA), D-sorbitol, maltodextrin, polyvinylpyrrolidone (PVP) 10, tremella polysaccharide (TP), and sucrose were purchased from Shanghai Aladdin Biochemical Technology Co., Ltd. (Shanghai, China). Staph Express Count Plate Petrifilm™ 6491 and Aerobic Count Plate Petrifilm™ 6406 were purchased from the Minnesota Mining and Manufacturing company (St. Paul, MN, USA).

### 2.2. Design of Cryoprotectant

#### 2.2.1. Preparation of *S. aureus*

*S. aureus* ATCC 25923 was initially stored at −80 °C. Before use, it was inoculated into nutrient agar culture medium and incubated at 36 °C for 18 h. After incubation, the colonies were collected and evenly dispersed in sterile water. The optical density (OD600) of the bacterial suspension at 600 nm was measured using a turbidimeter (DensiCHEK Plus, bioMérieux, Shanghai, China).

#### 2.2.2. Evaluation of Single Cryoprotectant

The impact of various cryoprotectants on the freeze-dried survival rate of *S. aureus* and physical appearance of the microsphere was evaluated. The conditions performed were the D-mannitol concentration (1, 2, 3, 4, 5% *w*/*v*), α-lactose concentration (2, 4, 6, 8, 10% *w*/*v*), sucrose concentration (4, 6, 8, 10, 12% *w*/*v*), TRE concentration (2, 4, 6, 8, 10% *w*/*v*), GLU concentration (2, 4, 6, 8, 10% *w*/*v*), SMP concentration (2, 4, 6, 8, 10% *w*/*v*), gelatin concentration (2, 4, 6, 8, 10% *w*/*v*), AG concentration (2, 4, 6, 8, 10% *w*/*v*), dextran 40 concentration (1, 2, 3, 4, 5% *w*/*v*), dextran 10 concentration (0.5, 1, 1.5, 2, 3, 4, 5% *w*/*v*), betaine concentration (2, 4, 6, 8, 10% *w*/*v*), PEG 20000 concentration (2, 4, 6, 8, 10% *w*/*v*), PEG 8000 concentration (2, 4, 6, 8, 10% *w*/*v*), Tween 40 concentration (0.02, 0.04, 0.06, 0.08, 0.1% *w*/*v*), glycine concentration (2, 4, 6, 8, 10% *w*/*v*), BSA concentration (1, 2, 3, 4, 5% *w*/*v*), D-sorbitol concentration (2, 4, 6, 8, 10% *w*/*v*), maltodextrin concentration (2, 4, 6, 8, 10% *w*/*v*), PVP 10 concentration (2, 4, 6, 8, 10% *w*/*v*), and TP concentration (2, 4, 6, 8, 10% *w*/*v*). After freeze-drying, the microsphere was re-dissolved in normal saline, and the bacterial count was recorded using the test piece reader. Using an electronic magnifier, the appearance of microspheres that contained different kinds of cryoprotectants was observed while testing their stability and solubility with a vortex. The survival rate was calculated according to Equation (1) [16].

(1) $$\mathrm{Survival}\;\mathrm{rate}\;(\%)=\frac{\mathrm{Viable}\;\mathrm{count}\;\mathrm{after}\;\text{freeze-drying}}{\mathrm{Viable}\;\mathrm{count}\;\mathrm{before}\;\text{freeze-drying}}\times 100\%$$

#### 2.2.3. Design of Composite Cryoprotectant

The response surface design allows for investigating the effects and interactions of variables. This design is more advantageous because it enables the optimization of multiple variables with a strongly reduced number of runs [17]. Based on the single cryoprotectant evaluation results, four kinds of cryoprotectants were selected for combination testing. A three-level four-factor Box–Behnken design was employed to optimize the cryoprotectant combinations [13]. The four factors and their high (1), medium (0), and low (−1) levels are shown in Table 1. Using Design-Expert 13, twenty-nine experiments were conducted (Table S1), and response surface diagrams were created to visualize the relationship between the freeze-dried survival rate of *S. aureus* and the interactions among the factors.

### 2.3. Process of Microsphere Preparation

The preparation of the *S. aureus* microsphere involves rapid molding followed by drying. All the cryoprotectants were sterilized at 121 °C for 15 min, excluding the protein components. A cryoprotectant was used to dilute the original bacterial suspension, then the pipettor was used to slowly drop 100 μL into the liquid nitrogen until the entire drop was frozen and sank (about 10 s) [15,18]. After that, the microspheres were clamped into the vial and placed into a freeze dryer (Epsilon 2-6D LSCplus, Marin Christ). Once the temperature stabilized, the first drying phase was initiated. The specific procedures reference earlier reports [11] and have been appropriately revised as follows: from −52 °C to −40 °C with a heating time of 40 min; −40 °C lasted for 24 h; from −40 °C to 25 °C with a heating time of 3 h; and 25 °C for 3 h.

### 2.4. Optimization of Microsphere Parameters

To investigate how the size of the microsphere affects the survival rate after freeze-dying, microspheres with volumes of 20, 40, 60, 80, and 100 µL were prepared, respectively. Similarly, to investigate the effect of *S. aureus* concentration on survival rate, microspheres with concentrations of 10^6^, 10^5^, 10^4^, 10^3^, 10^2^, and 10^1^ CFU were prepared, respectively. After freeze-drying, the microsphere was re-dissolved in normal saline. The number of bacteria in the microsphere was then determined using the Staph Express Count Plate Petrifilm™ 6491 after gradient dilution, and the survival rate was calculated.

### 2.5. Characterization of S. aureus Microsphere

The appearance of the microsphere surface and cross-section were observed using an electronic magnifier (50–1000×). A scanning electron microscope (SEM) (SU8020, HITACHI, Tokyo, Japan) and a transmission electron microscope (TEM) (JEM-1200EX, JEOL, Tokyo, Japan) were utilized to characterize the microstructure of *S. aureus* in three different states, namely before freeze-drying, freeze-dried without a composite cryoprotectant, and freeze-dried with a composite cryoprotectant [13]. Additionally, differential scanning calorimetry (DSC) (DSC25, TA Instruments, Newcastle, DE, USA) was employed to measure the glass transition temperature of the cryoprotectant [19]. The cell loss ratio of *S. aureus* was evaluated at a concentration of 100 mg/L lysozyme to assess cell wall damage [11].

### 2.6. Performance Evaluation of S. aureus Microsphere

#### 2.6.1. Moisture Content

Approximately 0.2 g of the microsphere was weighed, and the moisture content of the microsphere was determined using Karl Fischer Titration (917 Coulometer, Metrohm, Herrisau, Switzerland). The moisture content was measured at 0, 7, 14, 21, and 28 days after freeze-drying, respectively.

#### 2.6.2. Uniformity

Twelve freeze-dried *S. aureus* microspheres were randomly selected. The bacteria in each microsphere were determined after gradient dilution, and the coefficient of variation (CV) was calculated using Equation (2).

(2) $$\mathrm{CV}(\%)=\frac{\mathrm{Standard}\;\mathrm{deviation}}{\mathrm{Average}\;\mathrm{value}}\times 100\%$$

#### 2.6.3. Stability

The stability during storage was evaluated by storing them at three different temperatures, which were at 25 °C, 4 °C, and −20 °C. The number of bacteria within each microsphere was counted on days 0, 1, 3, 5, 7, 14, 21, 28, 60, and 90 following freeze-drying. Additionally, the appearance stability of the microsphere was assessed by subjecting them to vortex agitation for 60 s at speeds of 1000 rpm, 1500 rpm, and 2000 rpm. This process aimed to observe any potential breaking or partial detachment [18].

#### 2.6.4. Solubility

After freeze-drying, the microsphere was re-dissolved in normal saline. The solution was vortexed at 1500 rpm for 5 s and whether the microsphere completely dissolved was recorded.

### 2.7. Application of S. aureus Microsphere

According to existing reports on the method validation for the rapid detection of *S. aureus*, the feasibility of using the prepared *S. aureus* microsphere as a ready-to-use strain was evaluated based on the recovery rate [20]. Milk and green tea were chosen as the actual food samples and were purchased from the local supermarket. *S. aureus* was added to the milk and green tea at concentrations of 10^1^, 10^2^, and 10^3^ CFU/mL. The Staph Express Count Plate Petrifilm™ 6491 was then used to determine the number of bacteria in the milk and green tea samples. Finally, the recovery rate was calculated according to Equation (3) [21].

(3) $$\mathrm{Recovery}\;\mathrm{rate}\;(\%)=\frac{\mathrm{Detected}\;\mathrm{bacteria}\;\mathrm{number}}{\mathrm{Added}\;\mathrm{bacteria}\;\mathrm{number}}\times 100\%$$

### 2.8. Applicability of Preparation Process

The optimized composite cryoprotectant was utilized and the procedure described in Section 2.3 was followed. *E. coli* (ATCC 8739), *Salmonella* (ATCC 13076), *Shigella sonnei* (ATCC 9290), *Cronobacter muytjensii* (ATCC 51329), and *Klebsiella pneumoniae* (ATCC 13883) were chosen as the representative Gram-negative bacteria. *Listeria monocytogenes* (ATCC 19111), *Bacillus cereus* (ATCC 33019), and methicillin-resistant *S. aureus* (MRSA, ATCC 333591) were chosen as the representative Gram-positive bacteria, and MRSA was also the representative antimicrobial resistance bacteria. Furthermore, the potential use of mixed bacteria to address the current need for the simultaneous detection of multiple microorganisms was considered. Mixed solutions containing *S. aureus* with *Bacillus cereus*, *S. aureus* with *Cronobacter muytjensii*, and a mixed solution containing *S. aureus*, *Cronobacter muytjensii*, and *Bacillus cereus* were prepared. The Aerobic Count Plate Petrifilm™ 6406 was used to determine the number of bacteria before and after freeze-drying, and the freeze-dried survival rate was calculated using Equation (1).

## 3. Results and Discussion

### 3.1. Design of Cryoprotectant

#### 3.1.1. Evaluation of Single Cryoprotectant

**Figure 1:**
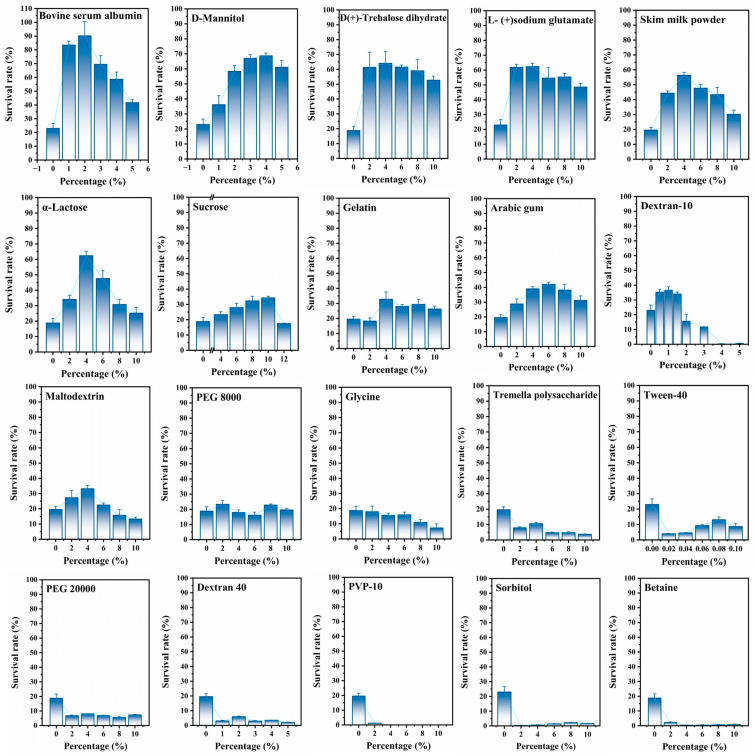
Different kinds of protective agents and their effects on the freeze-dried survival rate of *S. aureus* at different concentrations.

Different types of cryoprotectants have varying protective effects [22]. A typical freeze-dried formulation should contain several additional ingredients, including a bulking agent and protectant, alongside the active ingredients. Bulking agents provide mechanical rigidity to the freeze-dried products, preventing them from collapsing during the drying process. The protectant helps to stabilize the active ingredients against the stresses encountered during processing [23]. Based on the freeze-dried survival rate, the selection of the cryoprotectant should also consider the molding effect of the microsphere, as well as its appearance stability and solubility. Low concentrations of cryoprotectants may not effectively form a protective layer on the surface of bacteria. As the concentration of the cryoprotectant increases, the protective layer on the bacteria becomes denser, leading to improved protective effects. However, when the concentration exceeds a certain level, the components of the cryoprotectant may dehydrate the bacterial cells and damage their structures, resulting in a decreased survival rate [13,24]. As shown in Figure 1, the survival rate of *S. aureus* is approximately 20% in the absence of a cryoprotectant. The following single cryoprotectants: BSA (highest survival rate 90.2%), D-mannitol (highest survival rate 68.7%), TRE (highest survival rate 64.1%), GLU (highest survival rate 62.4%), SMP (highest survival rate 56.4%), α-lactose (highest survival rate 62.4%), sucrose (highest survival rate 34.3%), gelatin (highest survival rate 32.8%), AG (highest survival rate 42.1%), dextran 10 (highest survival rate 36.6%), and maltodextrin (highest survival rate 33.2%) were beneficial to improve the survival rate. Semipermeable cryoprotectants like TRE and sucrose can pass through the cell wall, and their free hydroxyl groups can form hydrogen bonds with the polar groups of proteins and phospholipids in the cell membrane, providing mechanical protection to the cells [25]. Impermeable cryoprotectants are macromolecules, such as BSA and SMP, that cannot penetrate the cell wall or membrane but offer protection from the outside. They can adsorb onto the outer surface of bacteria, forming a protective shell, creating a relatively sealed environment, preventing direct contact with ice crystals, and reducing exposure to oxygen, which minimizes potential damage [26]. In contrast, the following cryoprotectants: glycine, TP, Tween 40, dextran 40, PVP 10, D-sorbitol, and betaine were found to reduce the survival rate of *S. aureus*. In particular, dextran 40, PVP 10, D-sorbitol, and betaine resulted in survival rates lower than 10%, probably due to their bacteriostatic effects [27,28,29,30]. PEG 8000 did not significantly enhance or reduce the survival rate.

Some cryoprotectants result in droplet fragmentation upon freezing, resulting in an incomplete shape of the microsphere. As shown in Figure S1, BSA, PVP 10, AG, maltodextrin, gelatin, dextran 40, SMP, and TP negatively impact the molding of the microsphere, and D-sorbitol leads to significant shrinkage. The appearance of the microsphere surface and a cross-section with different cryoprotectants are shown in Figure S2. The surfaces of the microsphere that contain GLU and D-sorbitol are uneven, while the microspheres that contain gelatin, AG, TP, maltodextrin, PEG, and PVP exhibit various deformations. The cross-section of D-sorbitol shows multiple cavities, while cracks are evident in the cross-section of maltodextrin. Microspheres containing BSA, D-mannitol, TRE, SMP, α-lactose, sucrose, dextran, glycine, and betaine contribute positively to the formation of a regular microsphere. Under the conditions of vortex collision at 2000 rpm for 60 s, GLU, PEG 8000, PVP, dextran 40, and D-mannitol can maintain their spherical shape, although the powder shows varying degrees of degradation. In contrast, the microspheres containing gelatin, TP, and PEG 20000 remain unchanged, indicating a higher stability in appearance (Figure S3). The solubility of the microspheres is shown in Figure S4; upon adding normal saline, all the microspheres dissolved immediately except for TP and D-mannitol. After vortexing at 1500 rpm for 5 s, the solutions became transparent, with the exception of the TP. Therefore, despite TP being very effective for maintaining appearance stability, it cannot be considered the preferred component. Based on the above factors, BSA, TRE, PEG8000, and D-mannitol were selected for the further design of a composite cryoprotectant.

#### 3.1.2. Design of Composite Cryoprotectant

The freeze-dried survival rate was taken as the response value to further optimize the formula of the cryoprotectant. Taking the four factors (A, B, C, and D) as independent variables and the freeze-dried survival rate (Y) of *S. aureus* as a dependent variable, a three-level four-factor experiment was conducted (Table S1). The quadratic multiple regression equation was obtained as follows:

(4) Y = 100.43 − 3.82A + 0.15B − 2.01C − 0.70D − 2.57AB + 0.38AC + 1.04AD − 1.70BC + 3.94BD − 2.88CD − 6.71A^2^ − 10.28B^2^ − 3.99C^2^ − 6.15D^2^

As shown in Table 2, the F value of the model was 14.85, *p* < 0.0001, and the test result was significant. The F value of the lack of fit was 1.41, *p* = 0.3969, and the test result was not significant, indicating that the regression equation was well fitted. The model determination coefficient R^2^ = 0.9369 shows it can be used for the theoretical prediction and analysis of the freeze-dried survival rate of *S. aureus*. The results of the regression coefficient difference analysis of the model are as follows: A (BSA) is extremely significant, C (PEG8000) is significant, and B (TRE) and D (D-mannitol) are not significant. The interaction between B,D and C,D is significant (*p* < 0.05), and the interaction among AB, AC, AD, and BC is not significant. According to the F value, the influence of each factor on the freeze-dried survival rate of *S. aureus* is BSA > PEG 8000 > D-mannitol > TRE. The response surface diagrams are shown in Figure S5; compared with other factors, the response surface changes more steeply along the direction of BSA, demonstrating that BSA has the most significant influence on the freeze-dried survival rate of *S. aureus*, which is consistent with the analysis of variance.

Setting the aim response value as 100%, the composite cryoprotectant for *S. aureus* was predicted as follows: BSA 1.5%, TRE 4.5%, PEG 8000 8.2%, and D-mannitol 4.1%. Under these conditions, the survival rate of *S. aureus* was tested as 98.2 ± 2.6% after being repeated three times. The results showed that the optimized composite cryoprotectant was reasonable and feasible, and it can achieve excellent protection of *S. aureus*. Conventional freeze-dried bacterial powder usually requires several milliliters of cryoprotectant and cannot be sub-packaged. In contrast, freeze-dried microspheres only need 100 μL or even less, offering a significantly greater cost–benefit. Additionally, as shown in Table S2, the survival rate of *S. aureus* has increased by approximately 40% on average when comparing the composite cryoprotectant to three mature cryoprotectants, indicating an obvious improvement.

### 3.2. Optimization of Microsphere Parameters

As shown in Figure S6a, the freeze-dried survival rate of *S. aureus* ranged from 94.4% to 100.3 ± 4.0% when the volume of a single microsphere was between 20 and 100 μL, showing no significant difference (*p* > 0.05). Regarding the concentration of *S. aureus* in a single microsphere, Figure S6b demonstrated that the freeze-dried survival rate also showed no significant difference when the number of *S. aureus* in each microsphere ranged from 10^1^ to 10^6^ CFU (*p* > 0.05). These results indicate that neither the size of the microsphere nor the bacterial content within the microsphere significantly affects the survival rate. Thus, the parameters for the *S. aureus* microsphere can be tailored to meet specific needs.

### 3.3. Characterization of S. aureus Microsphere

**Figure 2:**
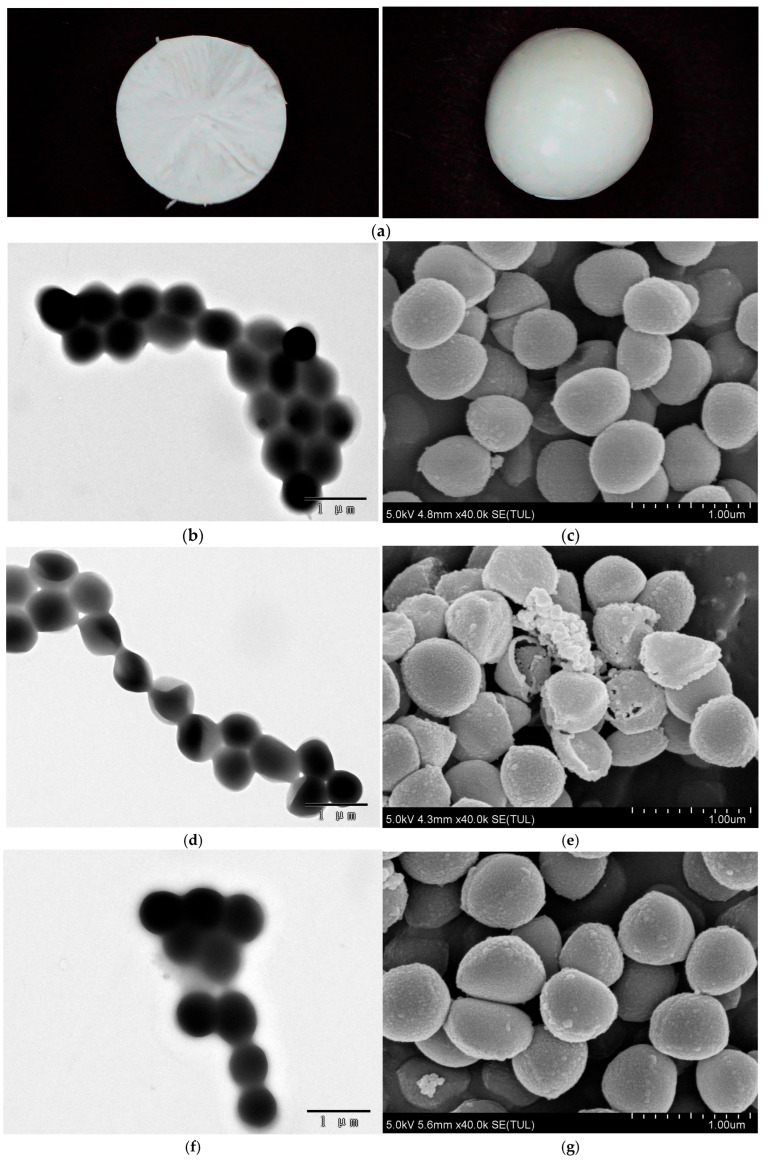
Characterization of *S. aureus* microsphere. (**a**) Appearance of microsphere surface and cross-section (diameter is about 5 mm); (**b**) transmission electron microscope and (**c**) scanning electron microscope characterization of *S. aureus* before freeze-drying; (**d**) transmission electron microscope and (**e**) scanning electron microscope characterization of freeze-dried *S. aureus* without cryoprotectant; (**f**) transmission electron microscope and (**g**) scanning electron microscope characterization of freeze-dried *S. aureus* containing composite cryoprotectant.

The glass transition temperature of the composite cryoprotectant measured by DSC was −23 °C. The appearance of the *S. aureus* microsphere surface and the cross-section formed by the composite cryoprotectant are shown in Figure 2a. The microsphere is regular and the surface is smooth, showing no cracks or cavities inside. TEM and SEM characterization of *S. aureus* without freeze-drying are shown in Figure 2b,c. The structure of bacteria appears intact and clear, maintaining a full and smooth shape, showing no cell damage and no content overflow. In the absence of cryoprotectants, the bacteria exhibit cytoplasmic shrinkage and noticeable cavities (Figure 2d), and this results in deformation. Additionally, the integrity of the cell wall membrane is compromised, resulting in the leakage of intracellular substances (Figure 2e). After using the composite cryoprotectant, the structural integrity of *S. aureus* was effectively preserved, remaining essentially the same as before freeze-drying (Figure 2f,g). This shows that the cryoprotectant effectively prevents *S. aureus* from shrinking, deforming, and damaging cell walls. The sensitivity of bacteria to lysozymes can be utilized to evaluate damage to the cell wall after freeze-drying [11]. As shown in Figure S7, the four types of protective agents could reduce the cell loss ratio compared to those without protectants, with BSA demonstrating the greatest effectiveness, followed by D-mannitol, TRE, and PEG. When BSA is combined with any other protective agent, there is a noticeable decrease in the cell loss ratio. Under conditions using the composite cryoprotectant, the cell loss ratio decreased to 2.2%, indicating that the cryoprotectants effectively maintain the integrity of the cell wall, thus facilitating a high survival rate of *S. aureus*, which is consistent with the results characterized by TEM and SEM. Overall, the results suggest that BSA is the most important component for preserving cell wall integrity during freeze-drying, consistent with findings that indicate that BSA yields the highest survival rate in single-cryoprotectant testing.

### 3.4. Performance Evaluation of S. aureus Microsphere

**Figure 3:**
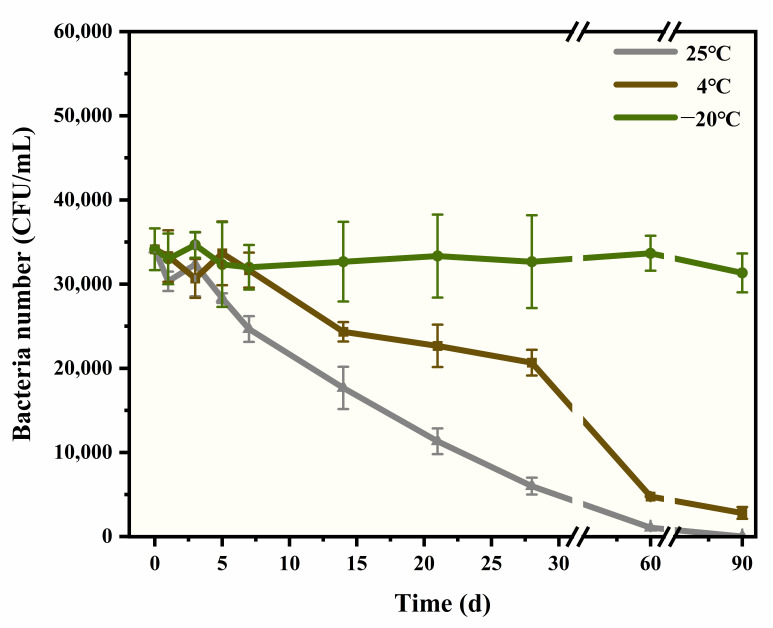
The bacterial number in *S. aureus* microsphere stored at 25 °C, 4 °C, and −20 °C within 90 days.

**Figure 4:**
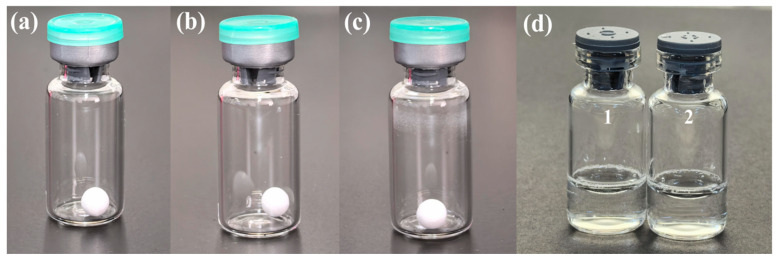
Appearance stability and solubility of *S. aureus* microsphere. (**a**) Vortex microsphere at 1000 rpm for 60 s; (**b**) vortex microsphere at 1500 rpm for 60 s; (**c**) vortex microsphere at 2000 rpm for 60 s; (**d**) microsphere dissolved by normal saline (1: without vortex; 2: vortex at 1000 rpm for 5 s).

When the moisture content of freeze-dried bacteria increases during storage, the bacteria’s “dormant state” resumes, and damage (such as lipid oxidation and protein degeneration) usually occurs, which leads to death [31]. As shown in Table S3, after freeze-drying, the moisture content of the microsphere is 0.153%, and the change is less than 0.01% within 28 days (storage at −20 °C), which is beneficial to stability. It is crucial to explore whether differences between individual microspheres will occur, thus affecting the repeatability and accuracy of the detection method. As shown in Table S4, the average number of *S. aureus* is 3.4 × 10^4^ CFU/each, and the CV is 7.1%, showing good uniformity. The storage temperature significantly affects the stability of *S. aureus*. As shown in Figure 3, according to the analysis of variance (ANOVA), at 25 °C, the bacterial number began to decline from the third day, and after five days, there was a significant reduction (*p* = 0.0179). The survival rate was only 17.6% by the 28th day. In comparison, at 4 °C, the bacteria exhibited better stability than at 25 °C, although there was still a significant decrease after 14 days (*p* = 0.0011). The survival rate was 60.5% by the 28th day. Under −20 °C, the best results were obtained, as there was no significant change over the 90 days (*p* > 0.05), with the survival rate exceeding 95%. Additionally, the microsphere demonstrated excellent appearance stability and solubility. As shown in Figure 4, there was no breaking or powder dropping after the vortex at 2000 rpm for 60 s. The microsphere dissolved immediately when normal saline was added, resulting in a transparent solution after just 5 s of vortexing.

### 3.5. Application of S. aureus Microsphere

The availability and reliability of the developed *S. aureus* microsphere as a ready-to-use strain for detection are shown in Table 3. The recovery rate for *S. aureus* in milk ranged from 80.0% to 86.9%, while in green tea, it ranged from 83.1% to 93.7% when the added concentration of *S. aureus* in the sample was between 10^1^ and 10^3^ CFU/mL. The coefficient of variation can be influenced by several factors, including instruments (such as pipettors and test piece readers), environmental conditions (such as temperature and humidity), and handling by personnel (such as dilutions). Thus, it is important to regularly calibrate instruments, monitor environmental parameters in real time, and implement standardized operating procedures. These results demonstrate that the microsphere is highly effective as a ready-to-use option for detecting *S. aureus* in actual samples.

### 3.6. Applicability of Preparation Process

The microsphere preparation for other foodborne pathogens showed notable differences in their survival rates. The results are shown in Table S5. The survival rates for Gram-negative bacteria, including *E. coli*, *Salmonella*, *Shigella sonnei*, *Klebsiella pneumoniae*, and *Cronobacter muytjensii* ranged from 34.1% to 75.2%. In contrast, the survival rates for Gram-positive bacteria, including *Listeria monocytogenes*, *Bacillus cereus*, and MRSA, were considerably higher, between 94.5% and 100.2 ± 5.9%. Current research indicates that it is nearly impossible that any cryoprotectant can equally protect all bacteria [13,32]. The strategy optimized in this study demonstrated a better protection effect on Gram-positive bacteria compared to Gram-negative bacteria; this may be due to the differences in the cell wall structure of the two types of bacteria [33,34]. In the future, a more suitable cryoprotectant for Gram-negative bacteria must be designed from existing cryoprotectants and freeze-drying technology to enhance the protection efficiency of these bacteria. Table S5 also presented the survival rates of mixed bacteria, which decreased by 21.6% on average compared to that of the single type. It was found that the more strains included, the lower the protective efficiency. The decrease in the survival rate of the mixed bacteria when freeze-dried may result from their differing affinities for the cryoprotectant. The formation of bonds or bridges between bacteria and the cryoprotectant may be influenced by the presence of other microorganisms. Increasing the concentration of the cryoprotectant may improve the survival rate, but systematic research is needed to achieve the most suitable results [35]. Therefore, further investigation into the interactions between mixed strains during the freeze-drying process is essential for advancing the development of the mixed microsphere in the future.

## 4. Conclusions

In order to develop an *S. aureus* strain for convenient and rapid use, this study designed a ready-to-use “ball-in-ball” microsphere based on a novel cryoprotectant combined with drop freeze-drying technology. When using the novel composite cryoprotectant, the survival rate of *S. aureus* can reach 98.2 ± 2.6%. The satisfactory recovery rate of *S. aureus* in milk and green tea further confirmed its feasibility and reliability in detecting actual samples. It has the advantage of convenient use, which can provide a more practical choice for improving detection efficiency and has potential as a general strategy for preparing ready-to-use strains related to food safety.

## Figures and Tables

**Table 1:** The factors contributing greatly to the *S. aureus* microsphere and their levels in Box–Behnken design.

Level	Factors			
	A: BSA (%)	B: TRE (%)	C: PEG 8000 (%)	D: D-Mannitol (%)
−1	1	2	6	3
0	2	4	8	4
1	3	6	10	5

**Table 2:** The results of response surface design and analysis of variance.

Source	Sum of Squares	df	Mean Square	F-Value	*p*-Value	
Model	1292.38	14	92.31	14.85	<0.0001	Significant
A	174.88	1	174.88	28.12	0.0001	
B	0.2821	1	0.2821	0.0454	0.8344	
C	48.56	1	48.56	7.81	0.0143	
D	5.95	1	5.95	0.9569	0.3446	
AB	26.52	1	26.52	4.27	0.0579	
AC	0.5929	1	0.5929	0.0953	0.7620	
AD	4.35	1	4.35	0.6991	0.4171	
BC	11.49	1	11.49	1.85	0.1955	
BD	61.94	1	61.94	9.96	0.0070	
CD	33.06	1	33.06	5.32	0.0369	
A^2^	291.82	1	291.82	46.93	<0.0001	
B^2^	685.29	1	685.29	110.21	<0.0001	
C^2^	103.45	1	103.45	16.64	0.0011	
D^2^	245.52	1	245.52	39.48	<0.0001	
Residual	87.06	14	6.22			
Lack of fit	67.79	10	6.78	1.41	0.3969	Not significant
Pure Error	19.27	4	4.82			
Cor Total	1379.43	28				

**Table 3:** Recovery rate of *S. aureus* in milk and green tea.

Sample	Added Level of *S. aureus* (CFU/mL)	Recovery Rate (%)
Milk	10^1^	81.5 ± 2.7
	10^2^	86.9 ± 1.3
	10^3^	80.0 ± 7.4
Green tea	10^1^	93.7 ± 3.2
	10^2^	83.1 ± 4.9
	10^3^	83.8 ± 4.4

## Data Availability

The original contributions presented in this study are included in the article/Supplementary Materials. Further inquiries can be directed to the corresponding author.

## Supplementary Materials

The following supporting information can be downloaded at: https://www.mdpi.com/article/10.3390/foods14122142/s1, Figure S1: *S. aureus* contains a single cryoprotectant that easily bursts when dropped into liquid nitrogen or shrinks after freeze-drying; Figure S2: The appearance of *S. aureus* microsphere surfaces and cross-sections freeze-dried with different cryoprotectants under an electron microscope; Figure S3: The changes in *S. aureus* microsphere appearance after colliding in a vortex; Figure S4: The solubility of the *S. aureus* microsphere freeze-dried by different cryoprotectants; Figure S5: Response surface diagram for the effect of the interaction; Figure S6: Optimization of microsphere parameters; Figure S7: The effect of the cryoprotectant on a cell loss ratio under 100 mg/L lysozymes of *S. aureus* after freeze-drying; Table S1: The survival rate of *S. aureus* in a response surface experiment based on Box–Behnken design; Table S2: The survival rate of *S. aureus* in the freeze-dried microspheres using other mature cryoprotectants; Table S3: Moisture content of *S. aureus* microsphere within one month after freeze-drying; Table S4: Difference in bacterial number in *S. aureus* microsphere; Table S5: Freeze-dried survival rate of single and mixed bacteria in microsphere using composite cryoprotectant.

## Abbreviations

The following abbreviations are used in this manuscript:

*S. aureus*	*Staphylococcus aureus*
*E. coli*	*Escherichia coli*
TRE	D(+)-Trehalose dihydrate
GLU	L-(+)Sodium glutamate
SMP	Skimmed milk powder
AG	Arabic gum
PEG	Polyethylene glycol
BSA	Bovine serum albumin
PVP	Polyvinylpyrrolidone
TP	Tremella polysaccharide
OD	Optical density
SEM	Scanning electron microscope
TEM	Transmission electron microscope
DSC	Differential scanning calorimetry
CV	Coefficient of variation
