# Supplementary material for: Design of Ready-to-Use “Ball-in-Ball” Staphylococcus aureus Microsphere Based on Novel Cryoprotectant and Drop Freeze-Drying Technology: Effective Preservation and Application
Source: Foods. 2025 Jun 19;14(12):2142. doi: 10.3390/foods14122142 (PMC12191541; doi:10.3390/foods14122142)
Supplement: Supplementary file 1 [file foods-14-02142-s001.zip › foods-3630176-supplementary.pdf]

Design of Ready-to-Use “Ball-in-Ball” *Staphylococcus aureus* Microsphere Based on Novel Cryoprotectant and Drop Freeze-Drying Technology: Effective Preservation and Application

Zile Wang [1], Dongdong Chen [1], Xiaomei Zheng [1], Yuqing Li [1], Shaoqian Jiang [1], Yanfei Chen [2], Jingjian Jia [1], Libo Yu [1] and Tao Peng [1,*]

[1] Chinese Academy of Quality and Inspection & Testing, Beijing 100176, China; jiangsq@acas.com.cn (S.J.)
[2] Eberly College of Science, The Pennsylvania State University, University Park, PA 16802, USA; zxt0616@163.com
* Correspondence: caiq_pengtao@126.com

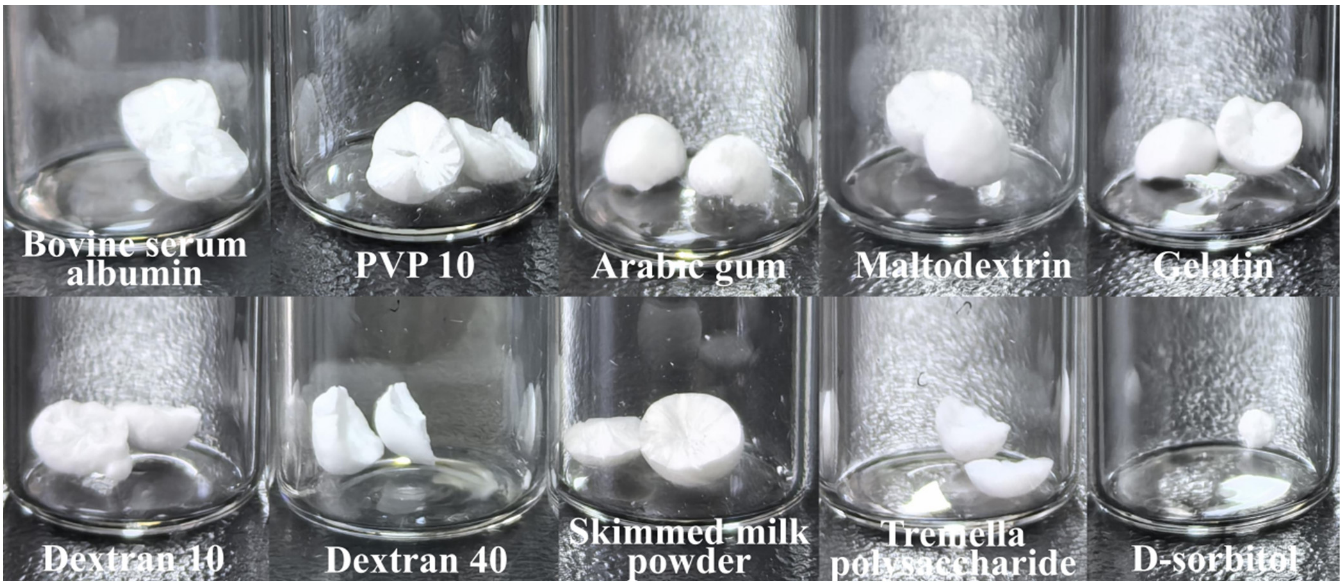

Figure S1 *S. aureus* contains a single cryoprotectant that easily bursts when dropped into liquid nitrogen or shrinks after freeze-drying.

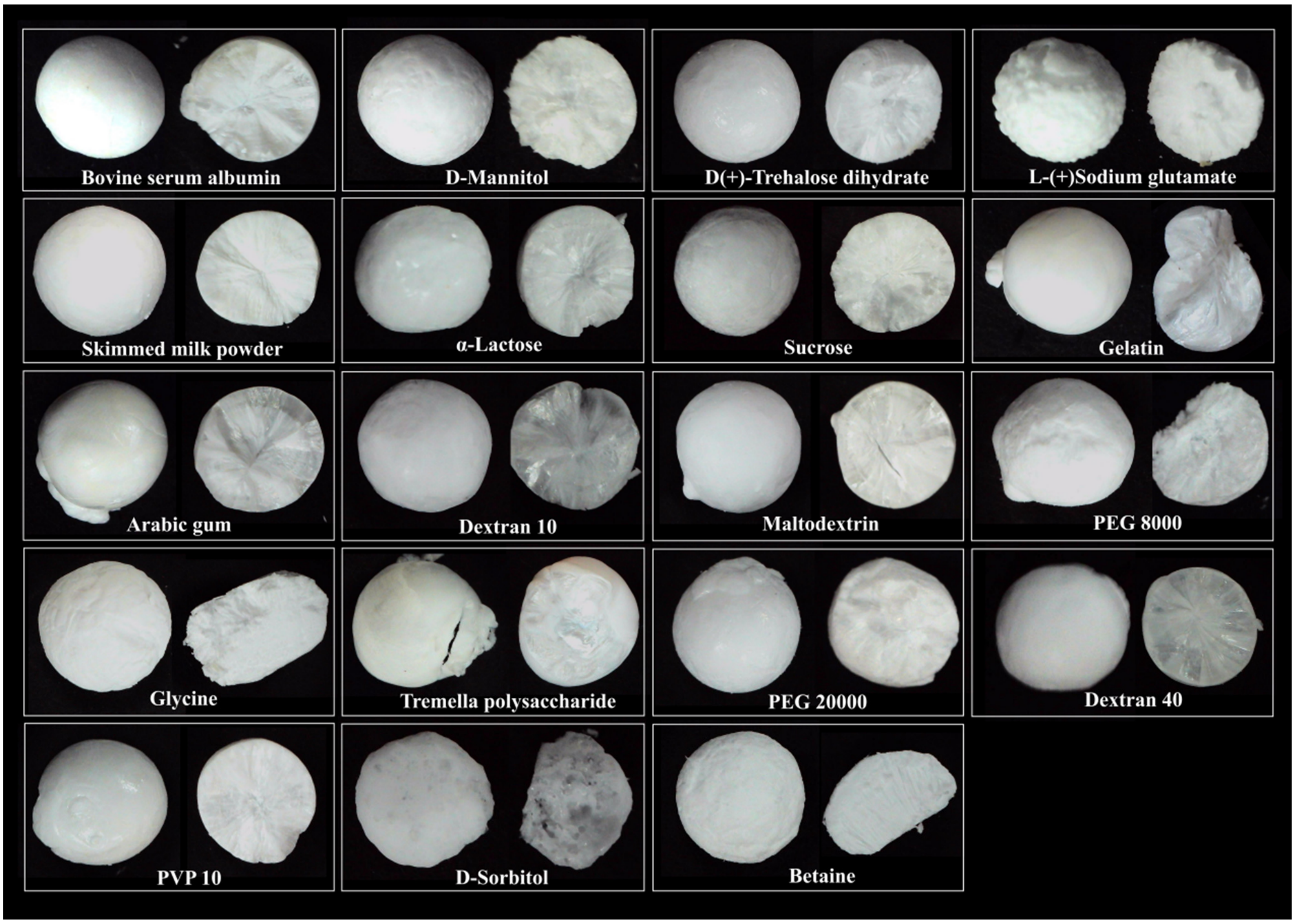

Figure S2 The appearance of *S. aureus* microsphere surfaces and cross-sections freeze-dried with different cryoprotectants under electron microscope.

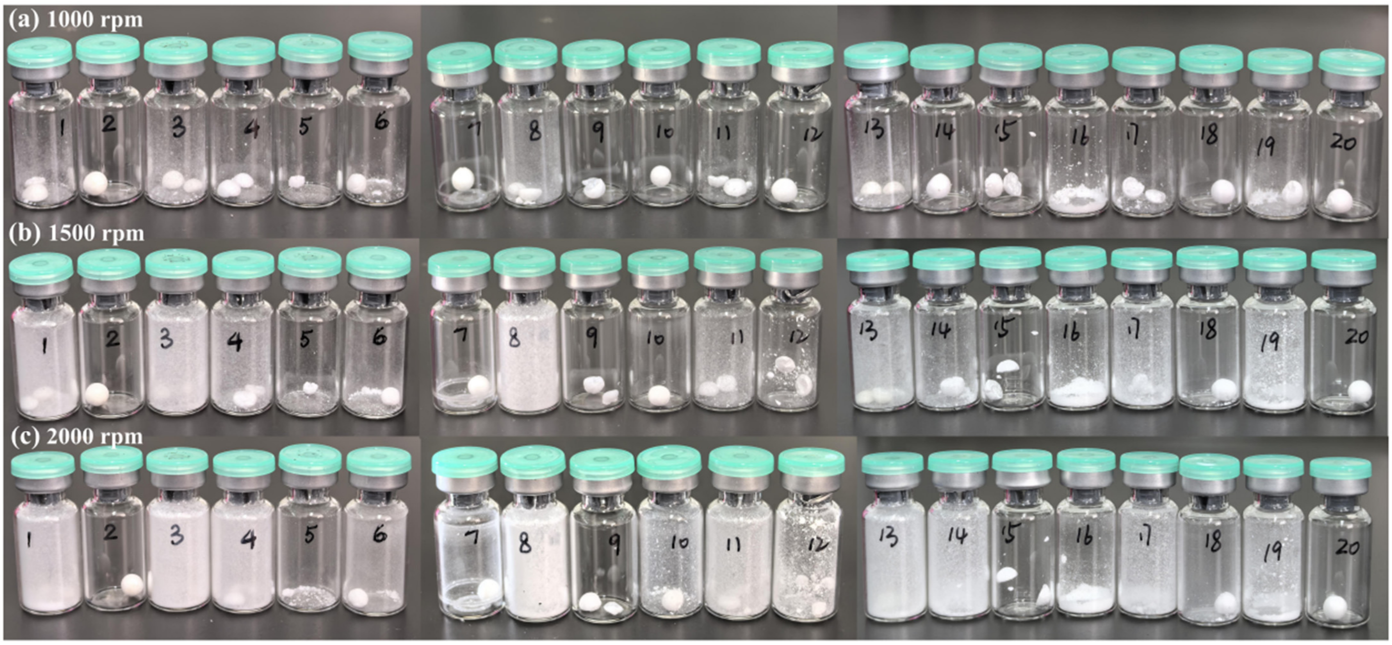

Figure S3 The changes of *S. aureus* microsphere appearance after colliding in a vortex (a) Vortex the microsphere at 1000 rpm for 60s; (b) Vortex the microsphere at 1500 rpm for 60s; (c) Vortex the microsphere at 2000 rpm for 60s (1: arabic gum; 2: gelatin;3: skimmed milk powder; 4: dextran 10; 5: D-sorbitol; 6: L-(+)sodium glutamate; 7: PVP K30; 8: PVP 10; 9: tremella polysaccharide; 10: PEG 8000; 11: maltodextrin; 12: sucrose; 13: bovine serum albumin; 14: D(+)-Trehalose dihydrate; 15: dextran 40; 16: glycine; 17: α-Lactose; 18: D-Mannitol; 19: betaine; 20: PEG 20000).

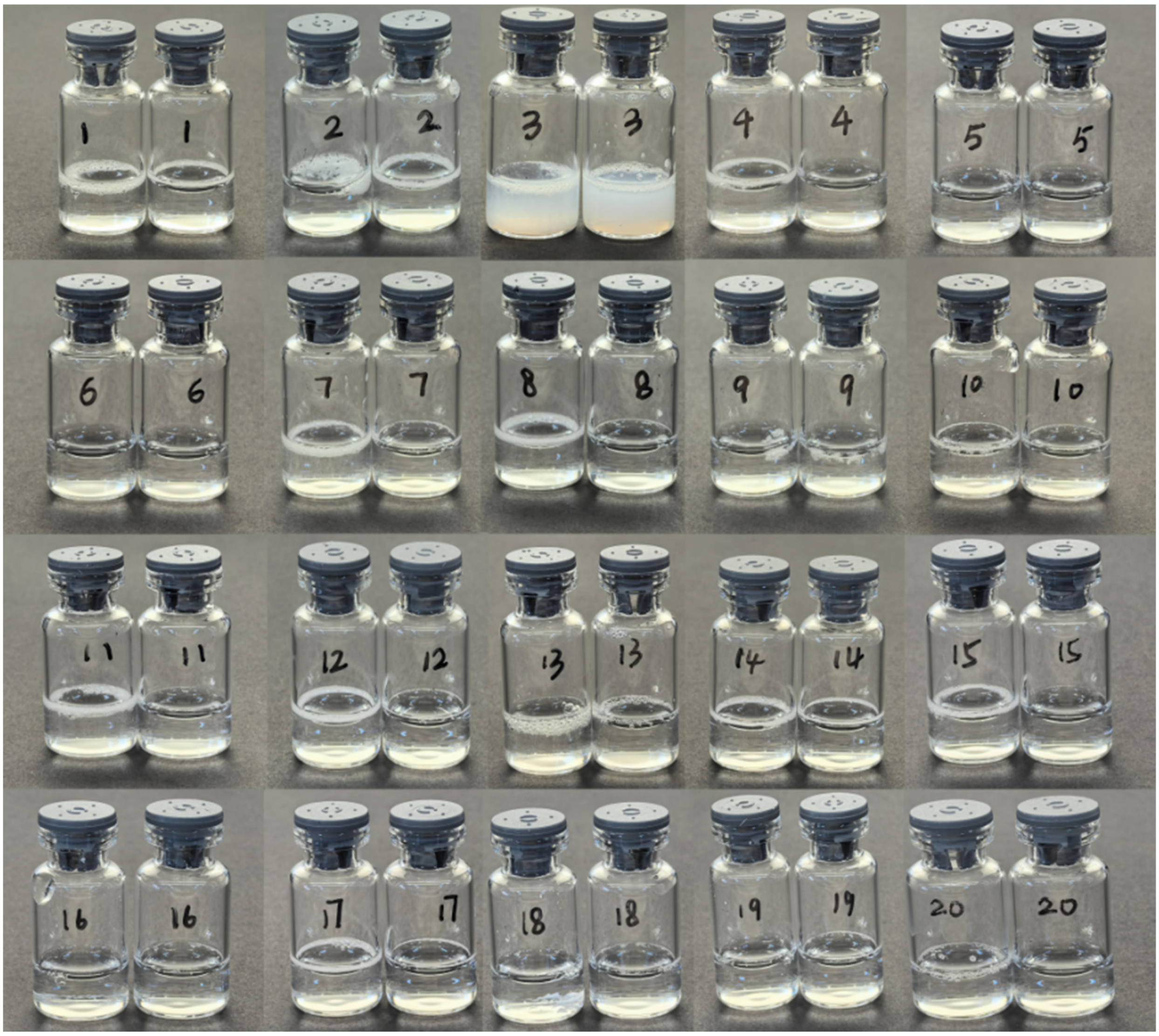

Figure S4 The solubility of *S. aureus* microsphere freeze-dried by different cryoprotectants (The left side is microsphere added normal saline without vortex, and the right side is microsphere added normal saline vortex at 1000 rpm for 5s. 1: arabic gum; 2: gelatin;3: skimmed milk powder; 4: dextran 10; 5: D-sorbitol; 6: L-(+)sodium glutamate; 7: PVP K30; 8: PVP 10; 9: tremella polysaccharide; 10: PEG 8000; 11: maltodextrin; 12: sucrose; 13: bovine serum albumin; 14: D(+)-trehalose dihydrate; 15: dextran 40; 16: glycine; 17: α-Lactose; 18: D-mannitol; 19: betaine; 20: PEG 20000).

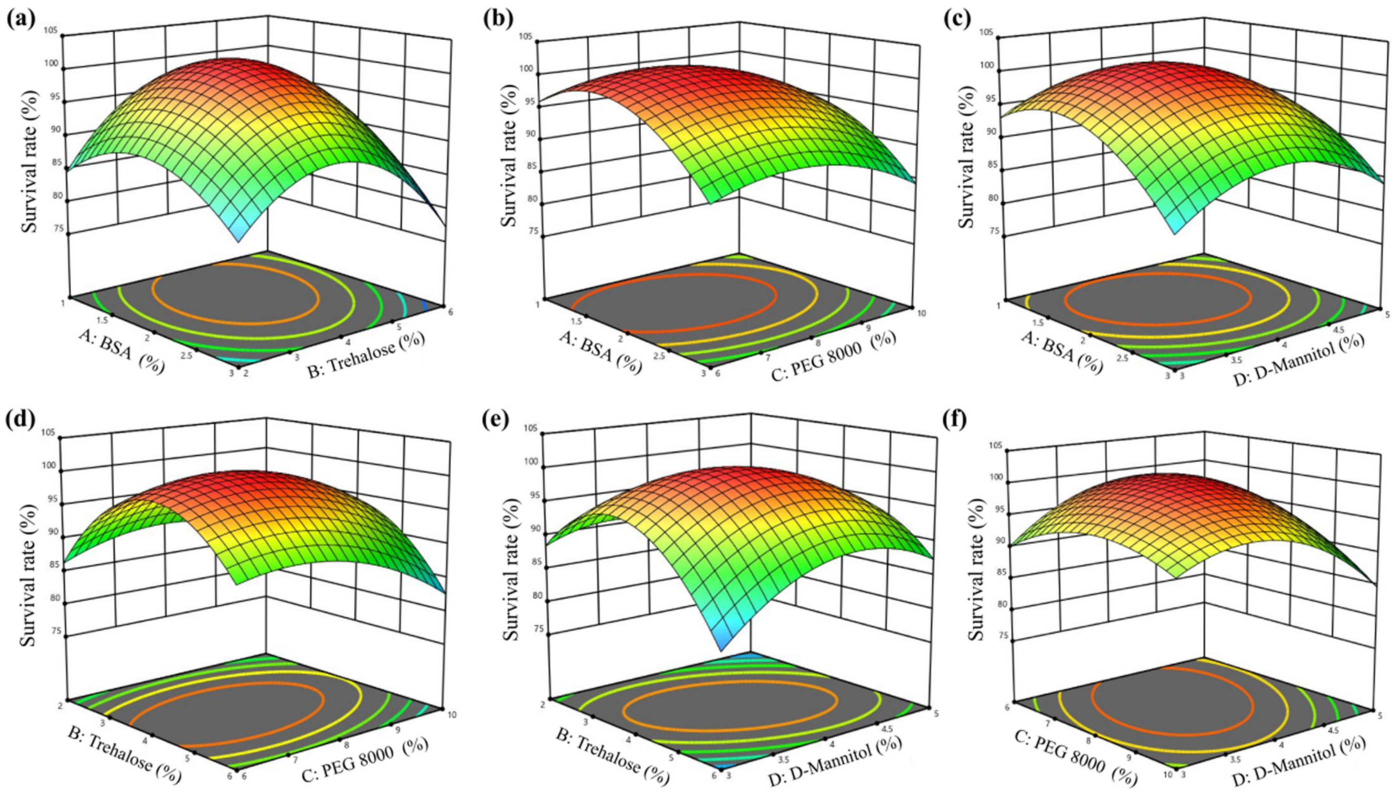

Figure S5 Response surface diagram for the effect of the interaction between (a) bovine serum albumin and trehalose (b) bovine serum albumin and PEG 8000 (c) bovine serum albumin and D-mannitol (d) trehalose and PEG 8000 (e) trehalose and D-mannitol (f) PEG 8000 and D-mannitol on the freeze-dried survival rate of *S. aureus*.

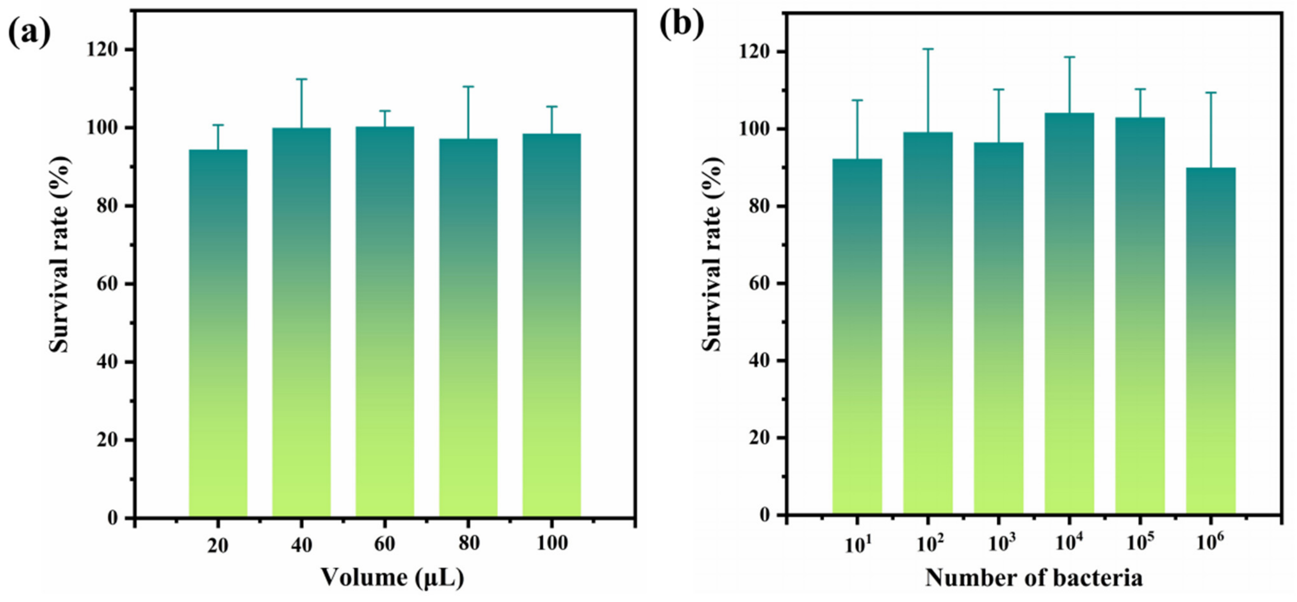

Figure S6 Optimization of microsphere parameters (a) The volume of a single microsphere; (b) The number of *S. aureus* in a single microsphere.

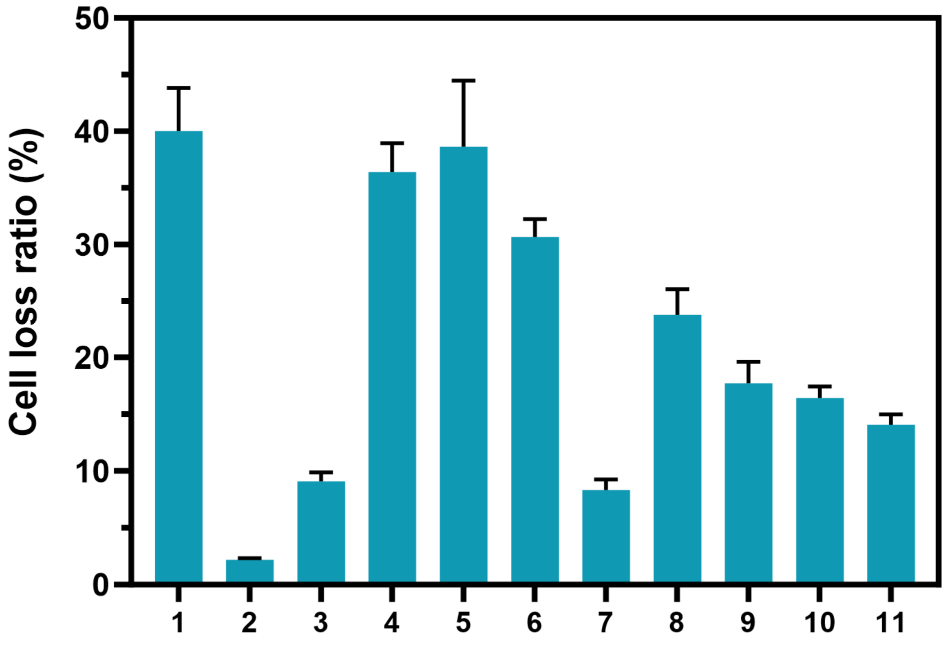

Figure S7 The effect of cryoprotectant on the cell loss ratio under 100 mg/L lysozyme of *S. aureus* after freeze-drying (1: without cryoprotectant; 2: bovine serum albumin, BSA; 3: D(+)-Trehalose dihydrate, TRE; 4: polyethylene glycol 8000, PEG; 5: D-Mannitol, MAN; 6: BSA+ TRE; 7: BSA+ PEG;8: BSA+ MAN;9: BSA+ TRE+PEG;10: BSA+ TRE+MAN;11: composite cryoprotectant).

Table S1 The survival rate of *S. aureus* in response surface experimental based on Box-Behnken design.

Run	Factor				Response
	A: BSA (%)	B: TRE (%)	C: PEG 8000 (%)	D: D-Mannitol (%)	Survival rate (%)
1	2 (0)*	6 (1)	8 (0)	5 (1)	86.15
2	2 (0)	2 (-1)	10 (1)	4 (0)	82.78
3	2 (0)	4 (0)	8 (0)	4 (0)	104.17
4	2 (0)	4 (0)	8 (0)	4 (0)	98.38
5	1 (-1)	2 (-1)	8 (0)	4 (0)	85.00
6	2 (0)	4 (0)	6 (-1)	3 (-1)	90.30
7	2 (0)	2 (-1)	6 (-1)	4 (0)	84.06
8	3 (1)	2 (-1)	8 (0)	4 (0)	85.30
9	2 (0)	4 (0)	8 (0)	4 (0)	99.59
10	2 (0)	4 (0)	8 (0)	4 (0)	100.00
11	1 (-1)	4 (0)	10 (1)	4 (0)	91.66
12	1 (-1)	4 (0)	6 (-1)	4 (0)	94.81
13	3 (1)	4 (0)	10 (1)	4 (0)	85.34
14	2 (0)	4 (0)	10 (1)	5 (1)	86.67
15	2 (0)	4 (0)	10 (1)	3 (-1)	91.03
16	3 (1)	4 (0)	8 (0)	3 (-1)	81.67
17	2 (0)	2 (-1)	8 (0)	5 (1)	79.49
18	2 (0)	6 (1)	8 (0)	3 (-1)	80.56
19	1 (-1)	4 (0)	8 (0)	5 (1)	89.30
20	1 (-1)	4 (0)	8 (0)	3 (-1)	94.72
21	2 (0)	6 (1)	10 (1)	4 (0)	82.78
22	3 (1)	6 (1)	8 (0)	4 (0)	78.89
23	2 (0)	6 (1)	6 (-1)	4 (0)	90.84
24	3 (1)	4 (0)	6 (-1)	4 (0)	86.95
25	1 (-1)	6 (1)	8 (0)	4 (0)	88.89
26	3 (1)	4 (0)	8 (0)	5 (1)	80.42
27	2 (0)	4 (0)	6 (-1)	5 (1)	97.44
28	2 (0)	4 (0)	8 (0)	4 (0)	100.00
29	2 (0)	2 (-1)	8 (0)	3 (-1)	89.64

* In parentheses are coded values.

Table S2 Survival rate of *S. aureus* in the freeze-dried microspheres using other mature cryoprotectants.

No.	Formula	Survival rate (%)
1	D(+)-Trehalose dihydrate 5% w/v, L-(+)Sodium glutamate 5% w/v, skimmed milk powder 5% w/v	80.0±3.0
2	Skimmed milk powder 10% w/v, D(+)-Trehalose dihydrate 14% w/v, glycerol 1.7% w/v	33.7±5.9
3	Skimmed milk powder 10% w/v, sucrose 10% w/v, gelatin 1% w/v, D-mannitol 1.5% w/v	57.9±1.5

Table S3 Moisture content of *S. aureus* microsphere within one month after freeze-drying.

Storage time (d)	Moisture content (%)
0	0.153
7	0.157
14	0.150
21	0.157
28	0.147

Table S4 Difference of bacterial number in *S. aureus* microsphere.

Microsphere	Bacteria (CFU/each)
1	33000
2	31000
3	36000
4	33000
5	37000
6	30000
7	35000
8	34000
9	34000
10	32000
11	36000
12	38000

Table S5 Freeze-dried survival rate of single and mixed bacteria in microsphere using the composite cryoprotectant.

No.	Bacteria	Survival rate (%)
1	*Escherichia coli*	51.5 ± 6.9
2	*Salmonella*	34.1 ± 3.0
3	*Shigella sonnei*	61.5 ± 5.3
4	*Klebsiella pneumoniae*	36.0 ± 4.1
5	*Cronobacter muytjensii*	75.2 ± 4.7
6	*Listeria monocytogenes*	98.2 ± 7.2
7	*Bacillus cereus*	100.2 ± 5.9
8	Methicillin-resistant *Staphylococcus aureus*	94.5 ± 11.3
9	*Staphylococcus aureus*	78.1 ± 6.6
	Bacillus cereus	85.5 ± 4.9
10	*Staphylococcus aureus*	93.8 ± 6.6
	Cronobacter muytjensii	74.4 ± 6.9
11	*Staphylococcus aureus*	73.7 ± 4.5
	Cronobacter muytjensii	39.9 ± 4.7
	Bacillus cereus	61.5 ± 7.3
